# Brain Death after Liver Transplantation for Fulminant Hepatic Failure: A Report of Two Cases

**DOI:** 10.5005/jp-journals-10018-1135

**Published:** 2015-01-06

**Authors:** Musa Akoglu, Ali Sürmelioglu, Ilter Ozer, Metin Ercan, Murat Ulas, Meral Akdogan, Erdal Birol Bostanci

**Affiliations:** 1 Department of Gastrointestinal Surgery, Turkiye Yuksek Ihtisas Education and Research Hospital, Ankara, Turkey; 2 Department of Gastroenterology, Turkiye Yuksek Ihtisas Education and Research Hospital, Ankara, Turkey

**Keywords:** Liver transplantation, Brain death, Fulminant hepatic failure.

## Abstract

Cerebral injury may persist and brain death may occur following an emergency liver transplantation (LT) for fulminant hepatic failure (FHF) even if the graft functions well. We herein present two patients developing brain death following emergency LT for FHF.

**How to cite this article:**

Akoglu M, Sürmelioglu A, Ozer I, Ercan M, Ulas M, Akdogan M, Bostanci EB. Brain Death after Liver Transplantation for Fulminant Hepatic Failure: A Report of Two Cases. Euroasian J Hepato-Gastroenterol 2015;5(1):62-64.

## INTRODUCTION

Fulminant hepatic failure (FHF) is a life-threatening condition, and the overall prognosis is quite poor without liver transplantation (LT). Emergency LT is the gold standard for the treatment of irreversible FHF.^1^ However, even after successful LT, cerebral damage may persist and brain death may develop despite favorable graft functions.^2^ In this case, the transplanted liver and the other organs may be reused for other recipients. However, successful reuse of transplanted liver has rarely been reported in the literature.^3^ In this paper, two cases of brain death following emergency LT were defined in whom the transplanted livers were subsequently harvested.

## CASE REPORTS

### Case 1

A 28-year-old male patient was admitted with a diagnosis of fulminant hepatic failure. Grade IV hepatic encephalo-pathy (HE) developed during his management and cranial computed tomography (CT) revealed minimal brain edema. Liver function tests were elevated with aspartate aminotransferase (AST), alanine aminotransferase (ALT) and total bilirubin levels being 2041 U/l, 1600 U/l and 17 mg/dl respectively. International normalized ratio (INR) was 7:3. After complete medical and detailed neurologic evaluation, national emergency call for LT was made in accordance with King’s College Criteria. Following an appropriate cadaveric donor being found from the national pool, LT was carried out without any peri-operative complication. In the postoperative period, although the patient was hemodynamically stable and his graft functions gradually improved (Table 1), he did not regain his consciousness. Doppler ultrasonography (USG) revealed normal hepatic arterial and portal venous flow. After 3rd postoperative day, polyuria and hyper-natremia (Table 1) developed, and neurogenic diabetes insipidus was considered. The liver enzymes continued to decrease and INR progressively improved; however, the patient still remained unconscious. Brain death was considered on the 5th postoperative day and detailed neurologic evaluation was performed. A cerebral scinti-graphy revealed absent blood flow and brain death was confirmed. Given well graft functions, reuse of the graft was considered. The patient’s relatives were asked for organ donation, and after their informed consent was obtained, his organs were submitted to the national organ pool. The kidneys were not accepted as they were horseshoe in shape. Fortunately, liver and heart were harvested and sent to different centers. The heart was evaluated and found not appropriate for transplantation; while the valves were harvested for valve replacement. Unfortunately, the center picking the graft from the pool decided not to reuse it due to their opinion that the hepatic artery had been damaged during harvesting.

**Table 1:** **Table 1:** Laboratory parameters of case 1

*Days*		*INR*		*ALT*		*AST*		*Bilirubin*		*Albumin*		*Sodium*	
1		1.92		826		927		13.1		2.71		145	
2		1.83		690		712		10.2		3.40		149	
3		1.49		527		599		7.9		3.56		154	
4		1.35		452		435		6.1		4.10		161	
5		1.21		412		350		4.5		3.97		172	

INR: International normalized ratio; ALT: Alanine aminotransferase; AST: Aspartate aminotransferase

### Case 2

A 30-year-old male patient was admitted with a diagnosis of FHF. Aspartate aminotransferase, ALT and total bili-rubin levels were elevated (1105 U/l, 2623 U/l and 23 mg/ dl respectively) and INR was 6.63. The patient developed grade 3 HE and cranial CT revealed minimal brain edema. After a complete medical and a detailed neurologic evaluation, emergency LT was planned in accordance with the King’s College Criteria. After an appropriate cadaveric donor was found from the national pool, emergency LT was performed. The patient did not regain consciousness although the liver functions gradually improved (Table 2). Doppler USG revealed normal portal vein and hepatic artery blood flow rates. After postoperative 3rd day, neurogenic diabetes insipidus with polyuria and hyper-natremia (Table 2) developed and appropriate medical treatment was begun. Given persisting unconsciousness, brain death was considered and a detailed neurologic evaluation was performed. Selective carotid vertebral angiography revealed the absence of cerebral blood flow, and brain death was confirmed on the 6th postoperative day. Because of a well-functioning graft, patient’s relatives were asked for organ donation. However, they refused.

## DISCUSSION

The outcomes of emergency LT for FHF are poorer than those of elective surgery. Postoperative mortality is mainly due to sepsis and multiorgan failure.^4^ In addition, although rare brain death may be a cause of death after emergency LT.^3^ In our two cases, reported in this paper, brain death developed in the early postoperative period, despite a well-functioning graft.

Currently, the selection criteria for the patients in emergency LT due to FHF are still controversial. In many centers in the world, the patients are picked with regards to King’s College and Clichy criteria.^5,6^ Grade III-IV HE is one of the most important prognostic sign among these criteria. The established certain contraindications for emergency LT include brain death, multiple organ failure and uncontrolled septic shock. However, the grade of HE is not regarded as a contraindication.^4^

In most of the grade III-IV HE patients, brain edema and intracranial hypertension are present.^7^ The brain damage caused by HE is usually reversible after LT.^5^ In controlling the brain edema and intracranial hypertension, the presence of a functional graft is considered as the most important factor.^7^ Nevertheless, in some grade III-IV HE patients, although the graft is functional, serious brain damage continue and brain death may develop consequently. In studies carried out in broad series, the rate of neurological deaths stated after emergency LT ranges between 4 and 22%.^1,2,8^ However, the reason of brain death is still not clear.

In the LT practice for FHF, one of the certain contraindications is brain death.^4,7^ Both of our cases had spontaneous ventilation; however, mechanical ventilator support was needed during the preoperative period. The brain stem functions were normal. In both patients, detailed neurological examinations were performed and brain death was excluded. However, in the post-transplant period, despite well-functioning grafts, brain death developed.

The first successful reuse of a liver graft practice was carried out in 1991, and it has been implemented in an increasing frequency so far.^3,9^ In all cases, the first recipients died of neurologic complications but were hemodyna-mically stable with evidence of well functioning liver grafts. Since, the grafts were well-functioning in our cases, patient’s relatives were asked about organ donation. In the first case, the heart and liver were ablated to be used and sent to another center upon the written consent.

In patients with FHF, a preoperative neurologic test predicting the occurrence of brain death in the post-transplant period is lacking. Liver transplantation will continue to be performed in accordance with the currently used criteria for the selection of patients, and it is inevitable that brain death can develop in some of these patients. We are of the opinion that the number of brain death cases following successful LT for FHF is much more than reported. Graft reuse from transplanted patients may still be regarded as a rare condition and our primary aim is to point out to the reuse of the well-functioning grafts when possible.

**Table 2:** **Table 2:** Laboratory parameters of case 2

*Days*		*INR*		*ALT*		*AST*		*Bilirubin*		*Albumin*		*Sodium*	
1		1.80		1257		2165		10.9		2.56		147	
2		1.57		701		748		7.2		3.86		151	
3		1.50		591		600		6.9		3.75		154	
4		1.40		548		487		6.7		4.14		157	
5		1.38		498		367		5.0		3.90		166	
6		1.26		340		220		4.1		3.56		178	

INR: International normalized ratio; ALT: Alanine aminotransferase; AST: Aspartate aminotransferase

## CONCLUSION

It should be kept in mind that brain death is possible despite successful LT and favorable graft functions. Patients who did not regain consciousness after LT should be evaluated for brain death. After confirmation of brain death, harvesting of the transplanted liver and the other organs should be considered.

## References

[1] Farmer DG, Dean M, Anselmo R (2003). Liver transplantation for fulminant hepatic failure: experience with more than 200 patients over a 17-year period.. Ann Surg.

[2] Bismuth H, Samuel D, Castaing D (1995). Orthotopic liver transplantation in fulminant and subfulminant hepatitis: the Paul Brousse experience.. Ann Surg.

[3] Nafidi O, Letourneau R, Willems BE (2007). Reuse of liver graft from a brain dead recipient.. Clin Transplant.

[4] Bernal W, Auzinger G, Dhawan A (2010). Acute liver failure.. Lancet.

[5] Bernuau J, Goudeau A, Poynard T (1986). Multivariate analysis of prognostic factors in fulminant hepatitis B.. Hepatology.

[6] O’Grady JG, Alexander GJ, Hayllar KM (1989). Early indicators of prognosis in fulminant hepatic failure.. Gastroenterology.

[7] Detry O, De Roover A, Honore P, Meurisse M (2006). Brain edema and intracranial hypertension in fulminant hepatik failure: pathophysiology and management.. World J Gastroenterol.

[8] Devlin J, Wendon J, Heaton N (1995). Pretransplantation clinical status and outcome of emergency transplantation for acute liver failure.. Hepatology.

[9] Moreno EG, Garcia GI, Gonzalez Pinto I (1991). Successful reuse of a livergraft.. Br J Surg.

